# Phytochemical profiling, antioxidant, enzymatic inhibitory, and antibacterial activities of *Wigandia ecuadorensis*

**DOI:** 10.3389/fpls.2024.1481447

**Published:** 2024-11-07

**Authors:** Rafael Viteri, Fernando Espinoza, Xavier Cornejo, Mario J. Simirgiotis, Patricia Manzano

**Affiliations:** ^1^ Centro de Investigaciones Biotecnológicas del Ecuador, ESPOL, Polytechnic University, ESPOL, Guayaquil, Ecuador; ^2^ Herbario GUAY, Departamento de Botánica, Facultad de Ciencias Naturales, Universidad de Guayaquil, Guayaquil, Ecuador; ^3^ Instituto de Farmacia, Facultad de Ciencias, Universidad Austral de Chile, Valdivia, Chile

**Keywords:** *Wigandia ecuadoriensis*, enzyme inhibition, antioxidant activity, antibacterial, phenols content, flavonoids

## Abstract

*Wigandia ecuadoriensis*, a member of the Namaceae family, is a source of metabolites and has been traditionally used as an anti-inflammatory. This work aimed to determine the total phenolic content (TPC), total flavonoid content (TFC), antioxidant effect, inhibition of α-glucosidase and cholinesterase enzymes (AChE, BChE), and antibacterial activity of the methanolic extract (ME) and subfractions of *Wigandia ecuadoriensis*. The findings revealed that ME and its subfractions exhibited significant antioxidant capacity, with the ethyl acetate fraction being the most active, displaying an IC_50_ of 17.66 µg/mL against the 1,1-diphenyl-2-picrylhydrazyl (DPPH) radical and 10.31 µg/mL against 2,2′-azinobis-(3-ethylbenzothiazoline-6-sulfonic acid) diammonium salt (ABTS). This activity was attributed to its high total phenolic content (357.47 mg GAE/g). Furthermore, *W. ecuadoriensis* fractions showed marked antimicrobial properties against human pathogen strains with Minimum Bactericidal Concentration (MBC) values ​​of 1.56–6.25 mg/mL for *S. aureus*, *E. faecalis* and *E. coli*. Furthermore, aqueous fraction exhibited slight inhibition of acetylcholinesterase (IC_50_: 915.98 µg/mL) and butyrylcholinesterase (IC_50_: 380.42 µg/mL). Interestingly, EF showed the greatest inhibitory effect of α-glucosidase (IC_50_: 38.44 µg/mL) which is more potent than the control used, acarbose (IC_50_: 179.07 µg/mL). UHPLC-QTOF-MS analysis identified forty compounds, including phenolic acids, flavonoids, saponins, terpenes, and fatty acyls. As far as we know, this is the first study to evaluate the chemical composition and biological potential of *W. ecuadoriensis*. Our results provide the first evidence to the chemical knowledge of the species *W. ecuadoriensis* and demonstrate its bioactive potential as an interesting source of secondary metabolites with possible beneficial properties for health.

## 1 Introduction

In recent years, there has been significant interest in using plant extracts. Indeed, it is recognized that plants harbor valuable bioactive compounds that can be beneficial both for promoting human well-being and for the formulation of supplements or nutraceuticals containing these enriching substances.

Antioxidant activity is essential to neutralize free radicals involved in aging and various chronic diseases, including cancer and cardiovascular diseases (Farhat et al., 2013). The study of inhibitors of enzymes such as α-glucosidase and cholinesterase is crucial in the search for valuable treatments for various diseases (Anand and Singh, 2013). Inhibition of α-glucosidase is an effective strategy in the management of type 2 diabetes mellitus, as it reduces blood glucose levels by slowing down carbohydrate digestion (Mustikasari et al., 2024). On the other hand, cholinesterase inhibitors play an important role in the treatment of Alzheimer’s disease, improving the transmission of nerve signals by preventing the breakdown of acetylcholine (Anand and Singh, 2013). Together, these studies provide a solid foundation for the development of therapies that combat metabolic and neurodegenerative diseases.

**Figure 1:**
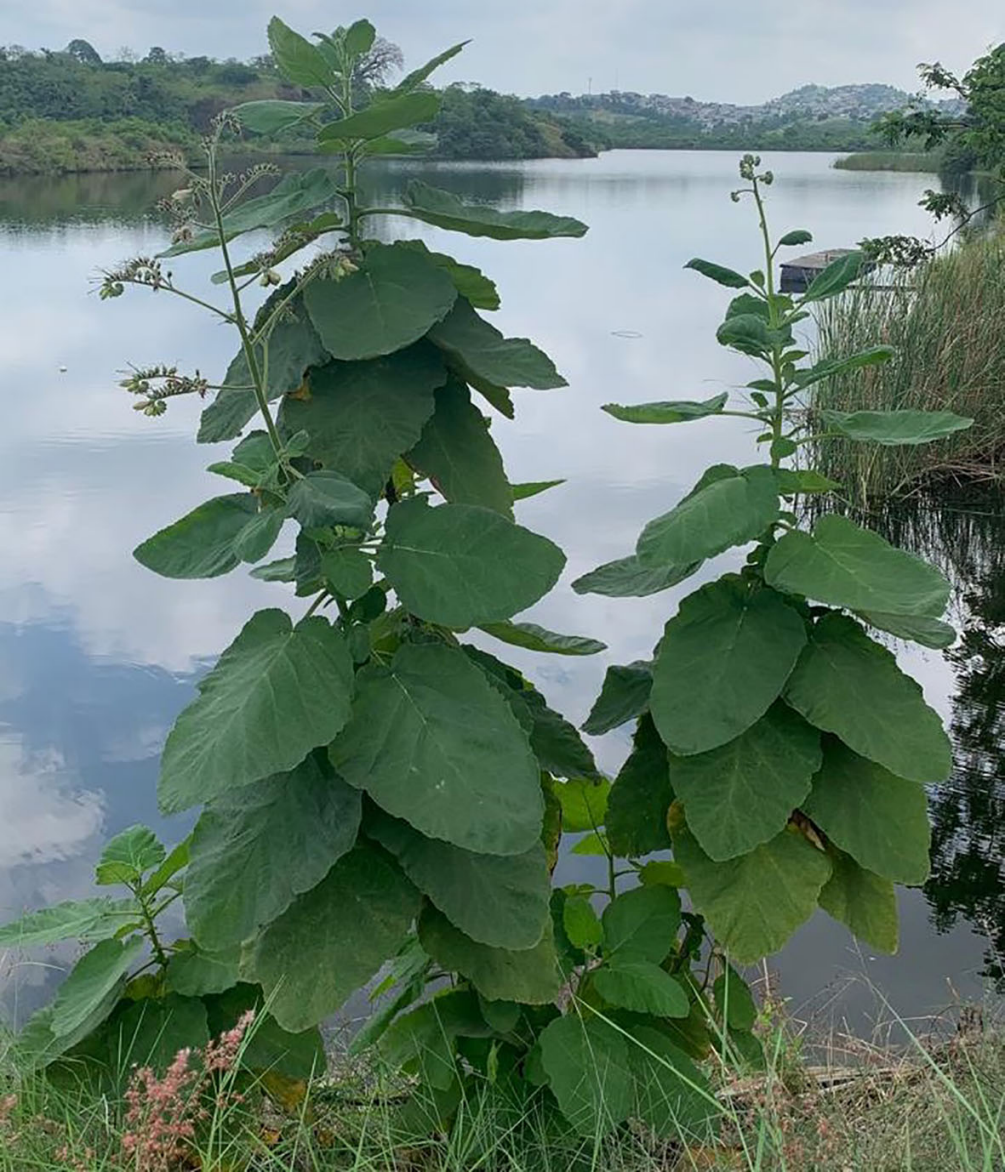
Wigandia ecuadoriensis.

In this sense, the Ecuadorian flora is known for its amazing diversity, hosting a unique wealth of plant species that arouse the interest of the scientific community. In this context, we focus on *Wigandia ecuadoriensis* (**Figure 1**), a new plant species botanically described in 2006 and which has captured the attention of botanists and ecologists due to its colonizing capacity and remarkable tolerance to reduced levels of precipitation, which could be used in the restoration of native vegetation in regions characterized by very dry tropical forests (Cornejo, 2006) and an interesting source of bioactive molecules. Although this species has been recognized locally, its detailed scientific study is still incipient.

*Wigandia ecuadorensis* is a shrub or small tree, up to 4 m tall, with large, shovel-shaped leaves and a terminal, branched inflorescence with pink flowers. It belongs to the Namaceae family and is endemic to the subtropical and tropical regions of the Ecuadorian coast (Cornejo, 2006; Vasile et al., 2020). In Ecuador, the Kichwa people used the leaves of the *Wigandia* Kunth genus as an anti-inflammatory (Torre et al., 2008). Other traditional uses of the *Wigandia urens* species are abortifacient, infections, epilepsy, psychological, skin, and immunological problems (Hitziger, 2016). The anti-inflammatory activity of different extracts of *Wigandia urens* has also been reported (Zavala-Sánchez et al., 2009). Some flavonoids and phacelioids have been isolated in the genus *Wigandia* (Gómez et al., 1980; Reynolds et al., 1989; Cao et al., 2003).

So far, there are no scientific studies in the literature on the plant species *W. ecuadorensis*, therefore, it represents an intriguing opportunity to discover new medicinal properties, unique chemical compounds, and possibly significant contributions to chemotaxonomy and natural medicine. This work aims to measure the content of phytochemicals (phenols and flavonoids) and evaluate the antioxidant, antibacterial, and enzymatic inhibition properties on cholinesterases and α-glucosidase of the methanolic extract and its subfractions from the leaves of *W. ecuadorensis*; and to analyze the metabolomic chemical profile by UHPLC-QTOF-MS of the fractions that presented the highest activity.

## 2 Materials and methods

### 2.1 Plant collection

The plant species *W. ecuadoriensis* was collected in Guayaquil, Guayas province, Ecuador (2°08’42.7”S 79°56’49.6”W) in October 2023. The species was identified by biologist Xavier Cornejo from the Faculty of Natural Sciences of the University of Guayaquil. A reference specimen (CIBE057) was deposited in the Herbarium of the Centro de Investigaciones Biotecnológicas del Ecuador, Guayaquil-Ecuador.

### 2.2 Extraction procedure and liquid–liquid fractionation

10 g of dried and powdered leaves of *W. ecuadoriensis* were macerated with methanol (three times with 100 mL each) under constant shaking using an orbital shaker (150 rpm) at 25°C for 24 h. Each extract was filtered through Whatman No. 1 filter paper and the solutions were concentrated under reduced pressure at 40°C to obtain the methanolic extract. Then, 2.9 g of the methanolic extract was suspended in 50 mL of a methanol-water mixture (1:3) and partitioned with *n*-hexane (HF), dichloromethane (DMF), ethyl acetate (EF), and a residual aqueous fraction (AqF). The extracts and subfractions were stored at -20°C until required for analysis.

### 2.3 Total phenolic content

Total phenolic content was determined according to the Folin-Ciocalteu (FC) assay (Ghareeb et al., 2017). 20 µL of the sample was mixed with 100 µL FC reagent (1:10 v/v) and 80 µL of a Na_2_CO_3_ solution (7.5%), incubated for 60 minutes at room temperature, and the absorbance of the resulting blue solution was measured at 760 nm in a microplate reader (Biotek Synergy HTX, Vermont, USA). The results of the total phenol content are expressed in mg of gallic acid equivalent per gram of dry extract (mg GAE/g DE). All measurements were carried out in triplicate.

### 2.4 Total flavonoid content

Total flavonoids were determined using the aluminum chloride method (Woster, 2003). 100 µL of sample was mixed with 100 µL of 2% AlCl_3_ solution in ethanol. After 60 minutes at room temperature, absorbance at 420 nm was measured using a microplate reader (Biotek Synergy HTX, Vermont, USA). The results of the flavonoid content expressed in mg of quercetin equivalent per gram of dry extract (mg QE/g DE).

### 2.5 Antioxidant assays

#### 2.5.1 DPPH radical scavenging assay

The antioxidant activity of extracts and subfractions was determined by the procedure described by (Thaipong et al., 2006). 50 µL of the sample was mixed with 150 µL of a DPPH solution (0.15 mM) dissolved in methanol in the dark for 30 minutes. Subsequently, the absorbance was measured at 517 nm in a microplate reader (Biotek Synergy HTX, Vermont, USA). A calibration curve with Trolox was used and the results were expressed in mg equivalent to Trolox/g DE.

#### 2.5.2 ABTS

The antioxidant capacity was measured through the iron reduction method described by (van den Berg et al., 1999). The radical ABTS stock solution is diluted to a final concentration of 156 µM to obtain a final absorbance of 0.70 ± 0.02 at 732 nm. The radical discoloration was initiated by adding 50 µL of the sample solution with 150 µL of the ABTS solution. After 30 minutes of incubation in the dark, the absorbance was measured at 732 nm using a microplate reader (Biotek Synergy HTX, Vermont, USA). The calibration curve was constructed with Trolox, and the results were expressed in mg equivalent to Trolox/g DE.

#### 2.5.3 Ferric-reducing antioxidant potential assay

The ferric reducing antioxidant power assay (FRAP) was determined according to the procedure described by (Ghareeb et al., 2017). In a 96-well microplate, 20 µl of each extract was mixed with 180 µl of FRAP reagent. The mixture remained for 30 minutes in the dark and the absorbance at 595 nm was measured in a microplate reader (BioTek Instrument, Inc., Winooski, VT, EE. UU.). The calibration curve was constructed with ferrous sulfate heptahydrate (FeSO_4_.7H_2_O), and the results were expressed in mmol Fe/g DE.

### 2.6 Cholinesterase inhibition

Acetylcholinesterase (AChE) and butyrylcholinesterase (BChE) inhibitions were performed *in vitro* according to the method of (Barrientos et al., 2023). The enzymes were dissolved in Tris-HCl buffer (50 mM, pH 8.0) and 5-dithiol-bis(2-nitrobenzoic) acid (DTNB) was prepared in buffer. *W. ecuadoriensis* fractions were prepared at a concentration of 20 mg per milliliter in buffer. 25 µL of the sample was mixed with 125 µL of DTNB and 25 µL of the enzyme (AChE 0.3 U/mL), incubated for 15 min at 37°C, then the substrates acetyl thiocholine iodide (15 mM) and butyryl thiocholine chloride (15 mM) were added, as appropriate, and the absorbance was measured at 412 nm at 37°C in a microplate reader (BioTek Instrument, Inc., Winooski, VT, USA). The results were expressed as IC_50_ values (μg/mL). Galantamine was used as a positive control.

### 2.7 α-glucosidase inhibition assay

To determine the inhibitory activity of α-glucosidase, 600 μL of phosphate buffer (100 mM pH 6.9) was added with 250 μL of *p*-nitrophenyl-α-d-glucopyranoside (5 mM), 100 μL of sample (EF/AqF from *W. ecuadoriensis*) and incubated at 37°C for 5 min. After that, 50 μL of 0.5 U/mL α-glucosidase enzyme solution was added to start the reaction. After 15 min at 37°C, 1000 μL of Na_2_CO_3_ (200 mM) was added. The absorbance was measured at 400 nm (Biotek Synergy HTX, Vermont, USA). The results were expressed as IC_50_ values (μg/mL). Acarbose was used as a positive control (Coral Caycho et al., 2020).

### 2.8 Determination of the minimum bactericidal concentration

A widely accepted sensitive serial dilution microplate method was used to determine the minimum inhibitory concentration (MBC) (Elisha et al., 2017). Overnight bacterial cultures were adjusted to McFarland standard 1, equivalent to 3.0 x 10^8^ CFU/mL (*Staphylococcus aureus*, *Enterococcus faecalis*, *Escherichia coli*, and *Pseudomonas aeruginosa*). The dried extract and subfractions were dissolved in 12.5% DMSO at a concentration of 25 mg/mL and 100 µL was added to the first well of a 96-well microtiter plate and serially diluted 1:1 with water. Bacterial cultures (100 µL) were added to each well. Starting with an extract concentration of 25 mg/mL, bacteria were therefore subjected to final concentrations of 6.25 to 0.05 mg/mL. Ampicillin was used as a positive control and DMSO (12.5%) as a solvent control. The highest concentration to which the bacteria were exposed was 12.5% DMSO in the first well and decreased two-fold in each subsequent well. Microplates were incubated overnight at 37°C. Finally, the solutions of the 96-well plates were subcultured in Petri dishes with 25 mL of soy agar. The MBC was defined as the lowest concentration of the extracts at which there was no sign of bacterial growth. The results are reported as mg/mL (Viteri et al., 2021). The antibiotic ampicillin was used as a positive control. For each bacteria and extract analyzed, a positive control (without plant extract) and a blank (without bacteria) were prepared.

### 2.9 UHPLC-QTOF-MS analysis

The separation and identification of the compounds present in the *W. ecuadoriensis* fractions was performed on a Compact QTOF MS + Elute UHPLC system, with the software Data Analysis 4.0 (all Bruker Daltonik GmbH, Bremen, Germany). Approximately 5 mg/mL of the fraction was dissolved in methanol and filtered through a 0.2 μm PTFE membrane and 3 µL was injected into the equipment. They were then measured in the chromatographic system consisting of a column temperature of 40°C; flow rate of 0.4 mL/min, mobile phase H_2_O + 0.1% formic acid (A) and acetonitrile + 0.1% formic acid (B), and elution gradient, 0–0.5 min (12% B), 0.5–11 min (1–99% B), 11–14 min (99% B), and 14–16 min (12% B). Mass spectrometry conditions: electrospray ionization (ESI) source, scanning range 50–1300 m/z, the fragmentation pattern was obtained using the spectral libraries of the MassBank of North America (MoNA), obtained from https://mona.fiehnlab.ucdavis.edu/downloads.

### 2.10 Statistical analysis

All the assays were performed in triplicate and represented as median ± standard deviation (SD) using Microsoft Excel software (Microsoft 365, Microsoft Corporation, Redmond, WA, USA). Statistical significance between groups was set at p < 0.05 and determined by one-way ANOVA with Tukey’s *post hoc* test using the commercial software Minitab 19.

## 3 Results and discussion

### 3.1 Extraction procedure

The total yield of the subfractions was performed after methanolic extraction and fractionation with *n*-hexane, dichloromethane, and ethyl acetate, resulting in four subfractions (hexane fraction, dichloromethane fraction, ethyl acetate fraction, and aqueous fraction). The results indicated that the methanolic extract (ME) yielded of 29.0%, followed by the aqueous fraction (AqF) which reached 15.5%. The dichloromethane fraction (DMF) had an intermediate yield of 8.5%, while the hexane fraction (HF) showed a much lower yield of 3.8%. Finally, the ethyl acetate fraction (EF) presented the lowest yield with only 1.0%.

### 3.2 Total phenolic and flavonoid content

The TPC and TFC of the extract and leaf fractions of *W. ecuadoriensis* were examined, and the results are presented in **Table 1**. The phenolic content was highest for the ethyl acetate fraction with 357.47 ± 12.78 mg GAE/g of dry extract. The flavonoid content was highest for the ethyl acetate fraction with 48.93 ± 6.32 mg QE/g of dry extract. Although this is the first report on *W. ecuadoriensis*, scientific information on the genus *Wigandia* and family Namaceae is scarce. However, there are some previous studies on other plants of the order Boraginales. The content of phenols and flavonoids in the ethanolic extract of *Eriodictyon californicum* leaves has been reported (Richards and Chaurasia, 2020). The authors report this species as a promising nutraceutical due to its healing properties against oxidative stress. Our results were superior to those reported for the species *Symphytum officinale* and *Anchusa ochroleuca*, with phenolic and flavonoid contents between 5.39-125.50 mg GAE/g of extract and 0.11-36.58 mg QE/g of extract, respectively (Trifan et al., 2021). In other species such as *Symphytum anatolicum* and *Cynoglottis barrelieri* the phenolic content was 32.7 and 52.8 mg GAE/g extract, respectively (Varvouni et al., 2020). In another study, it was determined that the phenolic and flavonoid content in the methanol extract of the *Onosma ambigens* species was lower with values of 51.19 mg GAE/g of extract and 45.39 mg QEs/g of extract, respectively (Sarikurkcu et al., 2020).

**Table 1:** Content of phenols, flavonoids of the different fractions of leaves of *W. ecuadorensis*.

Extract and fractions	Total Phenolic content (mg GAE/g DE)	Total Flavonoid content (mg QE/g DE)
ME	176.29 ± 8.87 ^b^	19.77 ± 4.53 ^b,c^
HF	43.13 ± 0.79 ^e^	26.89 ± 1.12 ^b^
DMF	88.93 ± 0.79 ^d^	9.51 ± 0.04 ^c^
EF	357.47 ± 12.78 ^a^	48.93 ± 6.32 ^a^
AqF	113.44 ± 7.94 ^c^	8.05 ± 0.07 ^c^

TPC, Total phenolic content; TFC, Total flavonoid content; Different letters in the same column indicate signiﬁcant differences: *p* < 0.05, *n= 3*.

### 3.3 Antioxidant activity

The antioxidant activity of the extract and subfractions was analyzed by DPPH, ABTS, and FRAP methods and are presented in **Table 2**. These methods are widely used due to their simplicity, sensitivity, and ability to provide comparative antioxidant capacities of various extracts and compounds (Tabart et al., 2009).

**Table 2:** Antioxidant activity of the different fractions of leaves of *W. ecuadorensis*.

Extract and fractions	DPPH (µmol TE/g DE)	DPPH (IC_50_ µg/mL)	ABTS (µmol TE/g DE)	ABTS (IC_50_ µg/mL)	FRAP (mmolFeSO_4_· 7H_2_O/gDE)
ME	179.80 ± 1.22 ^b^	35.70 ± 0.31 ^d^	183.88 ± 0.24 ^a^	30.19 ± 0.85 ^d^	2.27 ± 0.07 ^b^
HF	157.54 ± 0.49 ^d^	97.30 ± 2.44 ^a^	183.80 ± 0.37 ^a^	65.69 ± 1.34 ^a^	0.65 ± 0.01 ^e^
DMF	170.33 ± 1.52 ^c^	54.66 ± 1.28 ^b^	183.88 ± 0.42 ^a^	40.45 ± 0.57 ^c^	0.99 ± 0.03 ^d^
EF	185.82 ± 0.19 ^a^	17.66 ± 0.58 ^e^	183.96 ± 0.37 ^a^	10.31 ± 0.08 ^e^	2.87 ± 0.01 ^a^
AqF	187.32 ± 0.56 ^a^	41.14 ± 1.93 ^c^	184.12 ± 0.00 ^a^	62.36 ± 1.73 ^b^	1.69 ± 0.11 ^c^
Trolox *	n.a.	6.07 ± 0.46 ^f^	n.a.	5.55 ± 0.50 ^f^	n.a.
Ascorbic acid *	n.a.	3.06 ± 0.08 ^f^	n.a.	7.35 ± 0.18 ^e,f^	n.a.

*Used as standard antioxidant; DPPH, 2,2-diphenyl-1-picrylhydrazyl radical; ABTS, 2,2-azino-bis-3-ethylbenzothiazoline-6-sulfonic acid; FRAP, Ferric ion-reducing antioxidant power assay; Different letters in the same column indicate signiﬁcant differences: *p* < 0.05, *n= 3*; n.a, Not applicable.

**Figure 2:**
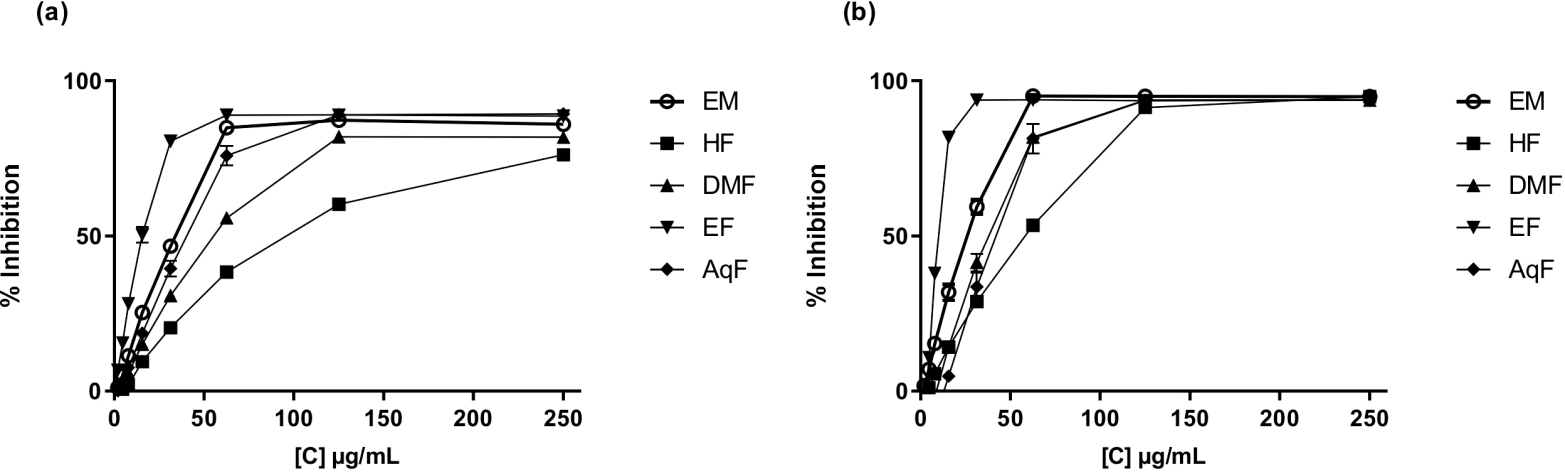
Antioxidant activity of *W. ecuadoriensis* extract and subfractions against DPPH **(A)** and ABTS **(B)**. Each point represents the average of three measurements.

The DPPH (2,2-diphenyl-1-picrylhydrazyl) assay measures the ability of antioxidants to scavenge free radicals by monitoring the color change from purple to yellow as the DPPH radical is reduced (Baliyan et al., 2022). The results of this assay ranged from 157.54 ± 0.49 to 187.32 ± 0.56 µmol TE/g of dry extract. *W. ecuadoriensis* ethyl acetate fraction scavenged DPPH in a concentration-dependent way with an IC_50_ of 17.66 µg/mL (**Figure 2A**). The hexane fraction had the highest value (IC_50_: 97.30 µg/mL). The antioxidant activity (IC_50_) decreased in descending order: EF > ME > AqF > DMF > HF. According to (Setha et al., 2013) IC_50_ values ​​< 50 µg/mL are considered potent antioxidants. A study reported that the extract of the *Eriodictyon californicum* species showed a 93.39% inhibition of DPPH radicals at a concentration of 1.0 mg/mL (Richards and Chaurasia, 2020), similar to those obtained in our study evaluated at the same concentration (ME: 85.97%, EF: 76.12%, DMF: 81.78%, EF: 88.63%, AqF: 89.30%). Likewise, a study reported that the extract of the polar aerial part of *S. officinale* showed a DPPH radical scavenging activity similar to our study (138.41 µmol TE/g) (Trifan et al., 2021). In *Cordia gilletii*, an IC_50_ between 3.2 - 83.5 µg/mL is reported in different extracts (Okusa et al., 2007).

The ABTS, 2,2’-azino-bis(3-ethylbenzothiazoline-6-sulfonic acid) assay involves the generation of a blue-green ABTS radical cation, which is reduced by the antioxidants present in the sample, resulting in a decrease in absorbance. Similarly, the antioxidant activity evaluated against the ABTS radical, the extract, and all fractions had similar values in the range of 183.80 ± 0.37 and 184.12 ± 0.00 µmol TE/g extract. *W. ecuadoriensis* ethyl acetate fraction scavenged DPPH in a concentration-dependent way with an IC_50_ of 10.31 µg/mL (**Figure 2B**). The antioxidant activity (IC_50_) decreased in descending order: EF > ME > DMF > AqF > HF. Likewise, the extract of the polar aerial part of *S. officinale* showed ABTS radical scavenging activity similar to our study (205.82 µmol TE/g extract) (Trifan et al., 2021). In another work it was observed that the Trolox equivalent antioxidant capacity (TEAC= IC_50_ Trolox/IC_50_ of the sample) ratio was 0.013, while in our study it was 0.18, concluding the antioxidant power of the methanolic extract of *W. ecuadoriensis* (Sarikurkcu et al., 2020).

The FRAP (ferric reducing antioxidant power) assay quantifies the antioxidant effect by evaluating the reduction of the ferric-tripyridyltriazine complex to its ferrous form, which has an intense blue color. FRAP values ranged from 0.65 ± 0.01 to 2.87 ± 0.01 mmol Fe/g of dry extract. The species of the genus *Eriodictyon* presented antioxidant activity with a concentration-dependent behavior comparable to the ascorbic acid standard (Richards and Chaurasia, 2022).

All these results demonstrate that the species *W. ecuadoriensis* and especially the ethyl acetate fraction present the highest antioxidant activity evaluated by the DPPH, ABTS, and FRAP methods, probably due to the higher total phenolic and flavonoid content compared to the other fractions tested (**Table 1**). In fact, the main compounds found in EF and AqF of *W. ecuadoriensis*, namely caffeic acids, rosmarinic acid, cirsimaritin, luteolin-7-glycoside and apigenin-8-C-(6”acetyl)-β-D-glucopyranoside (**Table 3**), have already been reported as DPPH and ABTS scavengers. In this sense, these results indicate that the compounds present in the EF and DMF fractions have a strong capacity to neutralize free radicals, suggesting a high bioactive potential. This antioxidant activity highlights the *W. ecuadoriensis* species as a promising source of natural antioxidants, which may have therapeutic and preventive applications in diseases related to oxidative stress.

**Table 3:** UHPLC-QTOF-MS identification of ethyl acetate (a) and aqueous fraction (b) from *W. ecuadoriensis* leaves.

No	T_R_(min)	Molecular formula	Major ion [M-H]^-^ (m/z)	Calculated Molecular Weight	Tentative Compound	Fraction
1	0.11	C_4_H_2_O_4_	112.9829	112.9856	Na formiate (internal standard)	EF, AqF
2	0.79	C_9_H_8_O_4_	179,0363	180,0436	Caffeic acid	AqF
3	1.08	C_21_H_20_O_12_	463,0859	464,0931	Hyperoside	EF, AqF
4	3.85	C_36_H_32_O_16_	719,1564	720,1641	Sagerinic acid	EF, AqF
5	4.59	C_21_H_20_O_11_	447,0928	448,1001	Luteolin-7-glucoside	EF
6	5.78	C_17_H_12_O_9_	359.0408	360.0494	Acetyl miricetin	EF, AqF
7	5,82	C_18_H_16_O_8_	359,0767	360,0832	Rosmarinic acid	EF
8	5.56	C_18_H_32_O_5_	327,2173	328,2246	Corchorifatty acid F	EF
9	5.78	C_18_H_32_O_5_	327,2173	328,2245	(10E,15Z)-9,12,13-trihydroxyoctadeca-10,15-dienoic acid	EF
10	6.19	C_17_H_14_O_6_	313,0695	314,0767	Cirsimaritin	EF
11	6.33	C_29_H_38_O_12_	577.2290	577.2574	Hydrangenoside C	EF, AqF
12	7.38	C_15_H_22_O_4_	265,1481	266,1554	Strobilactone A	EF
13	7.59	C_17_H_32_O_5_	315.2304	316.2329	Glyceryl-monomyristate	EF
14	7.77	C_28_H_44_O_7_	491.2803	492.2873	Hirsutalin C	EF, AqF
15	8.38	C_16_H_24_O_6_	311,1692	312,1765	Thymol-beta-D-glucoside	EF, AqF
16	8.81	C_18_H_32_O_3_	295,2271	296,2344	Dimorphecolic acid	EF, AqF
17	9.05	C_19_H_22_N_2_O	293,1784	294,185	Cinchonine	EF
18	9.22	C_23_H_22_O_12_	489.2627	490.2628	Luteolin-8-C-(6”acetyl)-β-D-glucopyranoside	EF
19	9.33	C_17_H_30_O_4_	297.2208	298.2223	Acaranoic acid	EF, AqF
20	9.47	C_17_H_30_O_4_	297.2198	298.2223	Acaranoic acid isomer	EF, AqF
21	9.8	C_17_H_28_O_4_	295.2054	296.1914	Acarenoic acid	EF, AqF
22	9.6	C_17_H_32_O_4_	299.2265	300.2227	Heptadecanedioic acid isomer	EF, AqF
23	9.32	C_23_H_22_O_12_	489.2802	490.2628	Luteolin-6-C-(6”acetyl)-β-D-glucopyranoside	EF, AqF
24	10.12	C_17_H_28_O_4_	295.2056	296.1914	Acarenoic acid isomer	EF, AqF
25	10.72	C_21_H_20_O_11_	447.0957	448.0933	Luteolin-6-C-β-D-glucopyranoside	EF
26	10.89	C_21_H_20_O_11_	447.0921	448.0933	Luteolin-8-C-β-D-glucopyranoside	EF, AqF
27	10.97	C_23_H_22_O_11_	473.2806	474.2797	Apigenin-6-C-(5”acetyl)-β-D-glucopyranoside	EF, AqF
28	11.14	C_23_H_22_O_11_	473.2798	474.2797	Apigenin-8-C-(6”acetyl)-β-D-glucopyranoside	EF, AqF
29	11.26	C_39_H_51_O_6_	615.3743	616.3691	Garcinol 13-O-methyl ether	EF, AqF
30	11.49	C_23_H_22_O_11_	473.2603	474.2797	Apigenin-8-C-(5”acetyl)-β-D-glucopyranoside	EF, AqF
31	11.85	C_32_H_54_O_10_	597.3644	597.3645	Kurilensoside G	EF
32	12.06	C_27_H_42_O_7_	477.2857	477.3078	Erinacine D	EF, AqF
33	12.24	C_19_H_27_	255.2111	255.2138	Unknown	EF
34	12.57	C_21_H_29_	281.2274	282.2285	Unknown	EF, AqF
35	12.72	C_25_H_39_O_4_	403.2853	403.2777	Uranediol diacetate	EF, AqF
36	13.45	C_32_H_52_O_9_	579.3538	579.3638	Tokoronin	EF
37	13.63	C_40_H_81_N_6_O_21_	981.5527	981.5527	Unknown	EF
38	14.23	C_21_H_40_O_7_	403.2701	403.2798	Aureosurfactin	EF
39	14.62	C_29_H_38_O_12_	577.2290	577.2574	Hydrangenoside C	EF
40	15.51	C_21_H_40_O_7_	403.2701	403.2798	Aureosurfactin	EF

### 3.4 Antibacterial activity of *W. ecuadoriensis*

In the study of the antibacterial activity of the extract and subfractions of leaves of *W. ecuadoriensis* against *Staphylococcus aureus*, *Enterococcus faecalis*, *Escherichia coli*, and *Pseudomonas aeruginosa*, it was found that the crude extract and the different fractions presented varied effectiveness (**Table 4**). The crude extract showed remarkable activity against *S. aureus* (1.56 mg/mL) and *E. faecalis* (3.13 mg/mL), being less effective against *E. coli*. Curiously, the hexane fraction was the most effective against *S. aureus* and *E. faecalis* (1.56 mg/ml), while the dichloromethane and ethyl acetate fractions presented limited activity. No activity was detected in the aqueous fraction. Ampicillin was used as a control, showing high effectiveness against *S. aureus* and *E. coli*. The genus *Wigandia* has been reported to have antimicrobial activities. The activity of three extracts (*n*-hexane, ethanol, and acetone) of *W. caracasana* leaves has been reported against the strains *Streptococcus pneumoniae*, *S. pyogenes*, *E. coli*, *Salmonella typhi* and *Shigella flexneri* with zones of inhibition between 6 and 12 mm (Cáceresa et al., 1993). Another species, *Wigandia urens*, has reported the antimicrobial activity of the ethanolic extract of leaves against strains *S. aureus*, *E. coli*, and *P. aeruginosa* with diameters between 13-19 mm (Rojas et al., 2003). Another study reported the antimicrobial activity of *Cordia oncocalyx* (Boraginaceae) with MIC values ​​<512 µg/mL (Thyalisson da Costa Silva et al., 2024). Our results were like those reported for the species *Echium humile*, who reported MCB values ​​between 1.56 and 12.5 mg/mL against S. aureus, 0.19 and 12.5 against E. faecalis, 1.56 and 3.12 against E. coli, using different extraction solvents (Aouadi et al., 2022). Although the extract and subfractions showed low antibacterial activity, these results are interesting, considering that these come directly from a leaf extract.

**Table 4:** Minimum bactericidal concentration (mg/mL) of leaf extracts against 4 pathogenic bacteria by microdilution assay.

Extract and fractions	*S. aureus*	*E. faecalis*	*E. coli*	*P. aeruginosa*
ME	1.56	3.13	6.25	–
HF	1.56	1.56	6.25	–
DMF	3.13	–	–	–
EF	6.25	6.25	–	–
AqF	–	–	–	–
Ampicillin *	2.06	–	0.26	–
Gentamicin *	0,19	1,56	0,39	6,25
Kanamycin *	0,78	6,25	1,56	–

*The antibiotic concentration is expressed as µg/mL (positive control). –, no inhibition.

### 3.5 Enzyme inhibitory activity

**Figure 3:**
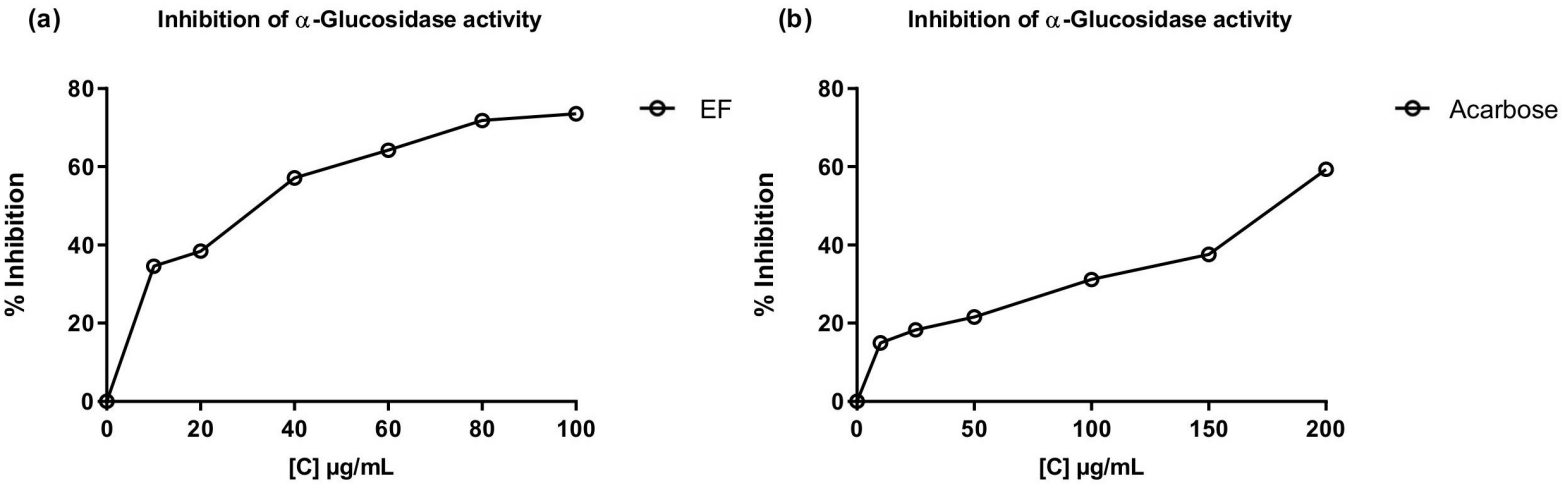
Inhibitory effects of EF from *W. ecuadoriensis* **(A)** and galantamine **(B)** on the enzyme α-glucosidase. Each point represents the average of three measurements.

The inhibitory activity of the most polar fractions (EF and AqF) of *W. ecuadoriensis* leaves was determined by spectrophotometric assays against α-glucosidase and cholinesterases (AChE, BChE) (**Table 5**). Inhibition of α-glucosidase is seen as an effective strategy for the control of obesity and diabetes (Zengin et al., 2018). This enzyme, located at the edge of the small intestine, breaks down complex carbohydrates into glucose. By inhibiting α-glucosidase, the metabolism of complex carbohydrates is slowed down, which lowers blood glucose levels (Mustikasari et al., 2024). **Figure 3** shows the effect of EF and acarbose on α-glucosidase enzyme activity. **Figure 3** shows that increasing the concentration of EF (10–100 µg/mL) and acarbose (10–200 µg/mL) increased the inhibition of α-glucosidase activity. At the highest concentrations, EF and acarbose (100 and 200 µg/mL) achieved inhibitions of 73.58 ± 0.36% and 59.39 ± 0.45%, respectively. The IC_50_ values ​​for α-glucosidase inhibition were 38.44 ± 0.75 µg/mL for EF (**Figure 3A**), which is approximately five times higher than the commercial standard, acarbose (179.07 ± 1.18 µg/mL, **Figure 3B**). These results indicate that the ethyl acetate (EF) fraction is more effective than acarbose in inhibiting α-glucosidase. According to (Benjamin et al., 2024) IC_50_ values ​​lower than 50 μg/mL are indicators of a strong potential as an inhibitor of α-glucosidase activity. In this sense, it is important to highlight that our result was obtained from a fraction and not from an isolated compound, which highlights a promising bioactive potential for the species. The presence of several unpurified compounds within the fraction suggests that upon further purification, the inhibitory activity could even be enhanced, revealing individual compounds with even stronger properties. This observed enzyme inhibition could be related to the presence of phenolic compounds described in **Table 1**. On the other hand, the aqueous fraction showed low inhibition, reaching only 4.75 ± 0.16% at a concentration of 2 mg/mL, therefore, its IC_50_ value could not be determined. Several studies have investigated the inhibitory potential of α-glucosidase in species of the order Boraginales, showing promising results. Another medicinal herb that has been used for centuries to treat diabetes is *Symphytum*. The inhibitory effect of the whole plant extract of *Symphytum anatolicum* showed a potent inhibitory activity (IC_50_: 18.28 ± 0.31 μg/mL), comparable to that exerted by acarbose (IC_50_: 17.05 ± 0.25 μg/mL), used as a control (Kılınc et al., 2023). The methanolic extract of *Echium humile* presented a value of 60 µg/mL, indicating a strong effectiveness (Aouadi et al., 2022). An *in vitro* antidiabetic study revealed that ethyl acetate extract exhibits 60% inhibition, at a concentration of 500 μg/mL, with an IC_50_ value of 380 μg/mL and the IC_50_ value of standard acarbose was 250 μg/mL (Syed Akbar et al., 2023). The aqueous extract of *Glandora diffusa* showed a potent inhibitory effect on a-glucosidase with an IC_50_ value of 33.3 µg/mL, almost ten times lower than that described for acarbose 300 µg/mL. These authors attribute this activity to the compounds caffeic acid and rosmarinic acid, which have been reported as inhibitors of α-glucosidase, and which are also present in our study (**Table 3**) (Ferreres et al., 2013). According to these IC_50_ values, the ethyl acetate fraction studied in this work seems to show an enzyme inhibition capacity comparable to that reported in previous studies.

**Table 5:** Enzyme inhibitory activity of *W. ecuadoriensis* fractions.

Fractions	α-glucoside		Cholinesterase inhibitory	
	% inhibition ± SD (2 mg/mL)	IC_50_ (µg/mL)	IC_50_ de AChE (µg/ml)	IC_50_ de BChE (µg/ml)
EF	85.83 ± 0.31 ^a^	38.44 ± 0.75 ^b^	–	–
AqF	4.75 ± 0.16 ^b^	–	915.98 ± 7.25 ^a^	380.42 ± 22.10 ^a^
Acarbose *	59.39 ± 0.45 ^c^	179.07 ± 1.18 ^a^	-	-
Galantamine *	n.a.	n.a	0.53 ± 0.03 ^b^	5.15 ± 0.44 ^b^

AChE, acetylcholinesterase; BChE, butyrylcholinesterase. Different letters in the same column indicate signiﬁcant differences: *p* < 0.05, *n= 3.* –, no inhibition; n.a, Not applicable. *Used as standard drug; Acarbose (0.2 mg/mL).

**Figure 4:**
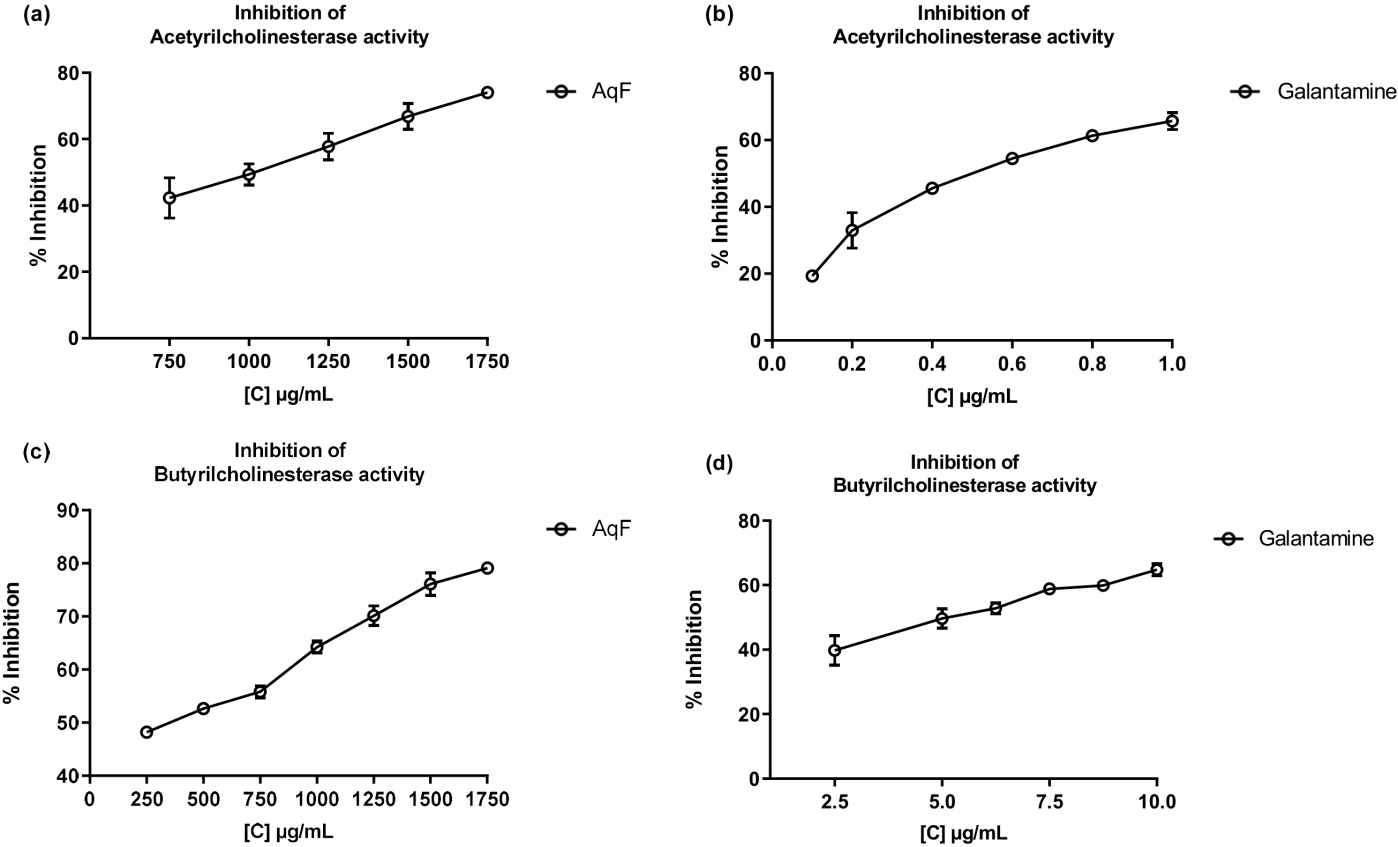
Inhibitory effects of AqF from *W. ecuadoriensis* and galantamine on cholinesterase enzymes. **(A, B)** Effect on AChE; **(C, D)** Effect on BChE. Each point represents the average of three measurements.

Cholinesterase inhibitors play a crucial role in the functioning of the nervous system and are related to the treatment of Alzheimer’s disease. Over the past two decades, the search for natural products related to AChE and BChE inhibition has increased (Ortega de Oliveira et al., 2024). **Figure 4** shows the inhibition of AChE and BChE enzymes in the presence of AqF and galantamine at increasing concentrations. The results revealed a dose-dependent behavior for the AChE enzyme with an IC_50_ value of 915.98 ± 7.25 µg/mL for AqF (**Figure 4A**). Regarding the effect on BChE, **Figure 4C** shows that the fraction inhibits the butyrylcholinesterase enzyme depending on the concentration with an IC_50_ value of 380.42 ± 22.10 µg/mL for AqF (**Figure 4C**). The ethyl acetate fraction did not show any inhibitory activity. These results were higher compared to the positive control galantamine, which presented an IC_50_ of 0.53 ± 0.03 µg/mL for AChE and 5.15 ± 0.44 µg/mL for BChE, indicating that the standard drug, galantamine, is more effective in inhibiting these enzymes compared to the aqueous fraction of *W. ecuadoriensis*. However, this result is still encouraging, since we are evaluating a fraction and not an isolated compound. It is likely that the bioactive compounds responsible for the activity are found in low concentration within the fraction, suggesting that further purification could significantly increase the inhibitory activity. The results obtained for AChE are comparable to that reported for the methanolic extract of *Wigandia urens*, with an AChE inhibition of 43% evaluated at a concentration of 1 mg/mL (Ortiz et al., 2013). There is information on the traditional use of the *Wigandia urens* species in Guatemala for epilepsy and psychological problems (Hitziger, 2016). On the other hand, some extracts from the Boraginaceae family (*S. anatolicum*, *S. aintabicum*, *Cynoglossum creticum*, *C. barrelieri*, and *Alkanna sfikasiana*) have shown inhibitory effects on AChE, BChE, and α-glucosidase (Varvouni et al., 2020). Our results presented lower IC_50_ compared to other species of *Onosma trapezuntea* and *Onosma rigidum* with IC_50_ values of 1270 and 1180 µg/mL for AChE and 2550 and 2060 µg/mL for BChE, respectively (Kirkan et al., 2022). Another study reported that hexane, chloroform, ethyl acetate and methanol extracts of *Calophyllum gracilentum*, at a concentration of 1.0 mg/mL, inhibited AChE by 3.02 ± 0.998%, 12.30 ± 5.641%, 31.62 ± 2.057% and 4.61 ± 2.129%, respectively (Seruji et al., 2024). In our study, we evaluated at the same concentration reported an inhibition of 51.20 ± 0.60%. Based on these values, the fraction investigated in this study seems to be more effective in inhibiting this enzyme compared to previously reported results.

The results obtained in this study reveal that certain fractions of *W. ecuadoriensis* possess a strong antioxidant potential and a remarkable inhibitory activity of the enzyme α-glucosidase, suggesting that this species could be a promising source of bioactive compounds. However, extensive research should be carried out to isolate the main compounds and determine their activity, in order to understand their mechanism of action better. Furthermore, these observations provide a valuable scientific contribution to the chemical knowledge and biological properties of the plant species *Wigandia ecuadoriensis*.

### 3.6 UHPLC-QTOF-MS analysis

**Figure 5:**
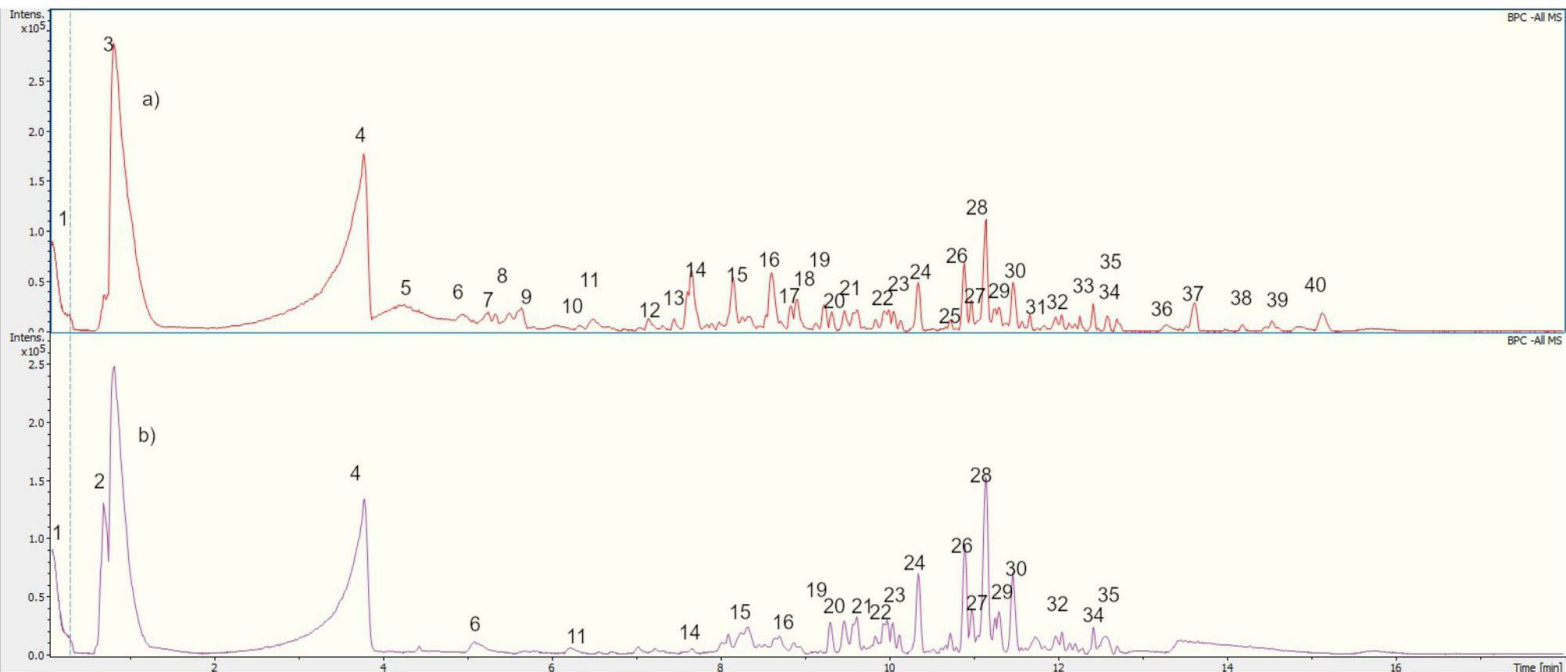
UHPLC-QTOF-MS Chromatogram of **(A)** EF and **(B)** AqF leaves *W. ecuadoriensis* in a negative ion mode.

The compounds from the ethyl acetate and aqueous fractions of the methanolic extract of *W. ecuadoriensis* leaves were analyzed by UHPLC-QTOF-MS. The total ion current chromatogram in negative ESI mode is shown in **Figure 5**, and the tentatively detected compounds are summarized in **Table 3**. The UHPLC-QTOF-MS profile revealed the presence of 39 metabolites, belonging to the classes of phenolic acids, flavonoids, fatty acyls, naphthofuran, glycerolipids, terpene, alkaloid, prenol lipids. The tentative identification was performed in Metaboscape software, a proprietary Bruker software that allows the identification of metabolites based on their mass, fragmentation pattern, and isotopic pattern, subsequently compared with the MassBank of North America (MoNA) database. These compounds include two phenolic acids (peaks 2 and 7), eleven flavonoids (peaks 3, 5, 6, 10, 18, 23, 25, 26, 27, 28 and 30), one lignan (peak 4), four fatty acyls (peaks 8, 9, 16, 22 and 38), four prenol lipids (peaks 11, 29, 32 and 39), one naphthofuran (peak 12), one glycerolipids (peak 13), two terpenes (peaks 14 and 15), and one alkaloid (peak 17), four lactones (peaks 19, 20, 21 and 24), three steroidal (peaks 31, 35 and 36), three unknown compounds (peaks 33, 34 and 37).

The compounds identified in the subfractions of *W. ecuadoriensis* exhibit a wide range of biological activities. Quinic acid shows antioxidant, antidiabetic, anticancer, antimicrobial, antiviral, antiaging, protective, antinociceptive, and analgesic properties (Benali et al., 2022). 3,4-Dihydroxybenzeneacetic and caffeic acids possess antioxidant activity in rat plasma (Raneva et al., 2001). Citric acid, known for its antimicrobial and antioxidant properties (Søltoft-Jensen and Hansen, 2005). Other compounds such as 3-hydroxybenzaldehyde, *p*-hydroxybenzoic acid, and hyperoside exhibit multiple properties, from antioxidant and anti-inflammatory to anticancer and organ protective (Rohini et al., 2013; Chen et al., 2021; Wang et al., 2022). Luteolin-7-glucoside, rosmarinic acid, and cirsimaritin have antioxidant, antitumor, anti-inflammatory, and protective activities against various diseases (Cai et al., 2020; Silva et al., 2020; De Stefano et al., 2021). Strobilactone A is known for its antifungal activity (Cohen et al., 2011), while thymol-beta-D-glucoside and dimorpholicacid have antibacterial activity (Mundt et al., 2003; Anderson et al., 2021). Cinchonina, in addition to being an antimalarial agent, has anticancer, antiobesity, anti-inflammatory, antiparasitic, antimicrobial, and antiplatelet effects (Parveen et al., 2024). Derivatives of acaranoic acid exhibit potent antifungal action against *Botrytis cinerea*, *Septoria tritici* and *Pyricularia oryzae* (Hussain et al., 2012). Luteolin-8-C-β-D-glucopyranoside and apigenin-8-C-(6”acetyl)-β-D-glucopyranoside stand out for their antioxidant capacity to scavenge free radicals (Simirgiotis et al., 2013). Kurilensoside G shows moderate inhibition in sea urchin sperm tests (Stonik et al., 2008). Erinacine D promotes nerve growth factor synthesis (Kawagishi et al., 1996). Tokoronin inhibits α-MSH-induced melanogenesis with low cytotoxicity (Ukiya et al., 2020). Aureosurfactin, a biosurfactant with comparable activity to rhamnolipid, surfactin and sophorolipid (Kim et al., 2016). Hydrogenoside C enhances cell viability and procollagen type I production in UVB-irradiated Hs68 cells (Shin et al., 2019).

## 4 Conclusion

This study is the first report of the *in vitro* activity of *W. ecuadoriensis* leaves. The ethyl acetate fraction was shown to have the highest content of phenols and flavonoids compared to the other fractions. This result elicited potent antioxidant activity. Furthermore, the ethyl acetate fraction was found to have strong potential as an inhibitor of α-glucosidase activity. On the other hand, the methanolic extract and its hexane fraction revealed antimicrobial activity. According to the UHPLC-MS results, the dominant compounds present in the fractions are caffeic acid, and hyperoside. These findings represent a valuable contribution to the knowledge of the species and suggest that *Wigandia* could be a promising source of bioactive compounds, creating new opportunities for the development of phytopharmaceuticals. Nevertheless, these initial results underline the need for further research to isolate the main compounds of the ethyl acetate fraction of *W. ecuadoriensis* to validate its antidiabetic effects.

## Data Availability

The datasets presented in this study can be found in online repositories. The names of the repository/repositories and accession number(s) can be found in the article/supplementary material.
